# Food Safety Training Is Associated with Improved Knowledge and Behaviours among Foodservice Establishments' Workers

**DOI:** 10.1155/2015/328761

**Published:** 2015-01-26

**Authors:** Hezekiah Kehinde Adesokan, Victor Oluwatoyin Akinseye, Grace Abiodun Adesokan

**Affiliations:** ^1^Department of Veterinary Public Health and Preventive Medicine, University of Ibadan, Ibadan 20005, Nigeria; ^2^Department of Health Education, University of Ibadan, Ibadan 20005, Nigeria

## Abstract

Though several studies have evaluated the association between food safety training and behavior, little has investigated different training components in association with food handlers' performance. Foodservice workers (*N* = 211) with at least two years' experience were willing to participate and were selected from major foodservice establishments in Ibadan, southwestern Nigeria, and completed a survey to evaluate the association between training, training area, duration, and refresher training and food safety knowledge and practices. We observed an association between training and knowledge (*P* = 0.000) as well as practices (*P* = 0.05) of food safety while different training areas contributed similarly to food handlers' knowledge (*P* = 0.17) and practices (*P* = 0.08). However, there was a significant decline in knowledge (*P* = 0.01) and practices (*P* = 0.001) with an increase in training duration. Furthermore, foodservice employees with refresher training demonstrated significantly higher knowledge (*P* = 0.000) and practice (*P* = 0.003) levels than those without, being about 45 and 14 times more likely to, respectively, improve their knowledge (OR = 45; 95% CI: 3.47–584.34) and practice (OR = 13.5; 95% CI: 2.01–90.69). Researchers should always consider varying training components before making assertions regarding effectiveness of training on foodservice workers' behaviour.

## 1. Introduction

The incidence of food-borne diseases has been on the increase, often associated with outbreaks, and threatens global public health security and raises international concern [1]. The World Health Organization reported that 1.8 million deaths in 2005 alone resulted from diarrheal diseases, most of which were attributed to the ingestion of contaminated food and drinking water [2]. According to the Center for Disease Control and Prevention [3], 59% of food-borne disease outbreaks involved foodservice establishments. Previous reports [4, 5] indicated that poor food handling practices are a leading cause of food-borne diseases. Such improper practices have been well documented [6] and typically include cross contamination of raw and cooked food, inadequate cooking, and storage at inappropriate temperatures. Food handlers may also be asymptomatic carriers of food poisoning organisms [7], serving as a potential source of contamination to food. However, adequate training and transfer of such training to behaviour in particular can help limit such improper food handling practices and hence reduce the resulting effects of contamination on health and economy.

Food handler training is seen as one strategy whereby food safety can be increased, offering long-term benefits to the food industry [8]. Previous findings suggest that while food safety training might increase knowledge [9], the knowledge might not always translate into improved behaviours [10]. Such transfer problem has been linked to a number of factors including trainee characteristics, training design, and work environment [11–13]. Hence, several studies conducted on the effectiveness of food safety training on behaviour in foodservice establishments yielded inconsistent conclusions; many studies found that training was effective [10, 14, 15] while others drew the opposite conclusion [16, 17].

In practical terms, the length and areas of food safety training received often differ from one food handler to the other and hence might result in differing levels of output. While some might have received shorter training in a specific area of food safety, others might have attended much longer training in the same or other areas and the ability to cope with short or long training also differs. Similarly, food safety performance of trained food handlers with additional regular refresher training might be quite different from those who only had training once. The purpose of this study is therefore to examine the association between self-reported training, areas of training, training duration, and refresher training and the knowledge as well as practices of food safety among foodservice workers in Ibadan, southwestern Nigeria. This is to provide an important step in increasing our understanding regarding the inconsistent reports by previous studies on the effectiveness of training on food handlers' behaviour.

## 2. Materials and Methods

The study was conducted in Ibadan, southwestern Nigeria. Ibadan is a cosmopolitan city characterized by increasing outdoor or street food consumption despite pockets of cases of food-related diseases. Self-administered questionnaires were completed in the presence of the researchers by 211 foodservice workers who were selected based on the inclusion criteria of serving as foodservice workers for at least two years and were willing to participate in the study. These foodservice workers were from different indoor foodservice establishments dealing in processed and ready-to-eat food as well as raw unprocessed foods in permanent locations in the area. They were involved in different food processing stages ranging from handling raw food, food preparation, and cooking to packaging and sales. The establishments ranged from staff strength of 5–10 to 20 and above. The content validation of the questionnaires was done by cross-reference (Cronbach's alpha = 0.7743) and verification from food safety experts who have been trained in the field and understood the foodservice systems of the study area with better acquaintance with the foodservice industry. A pretest was carried out by the authors among ten foodservice workers after which some of the questions were modified in order to improve clarity. Oral consent to participate in the study was obtained from each respondent. The questionnaires examined the association between self-reported training, area of training, training duration, and refresher training and food safety knowledge and practices of the food handlers. The respondents' sociodemographic characteristics, such as age, gender, food safety training, area of training, training duration, and refresher training received, were documented during the study. They were classified based on age groups <20 years, 20–40 years, and >40 years; education status: no formal education, primary education, and postprimary education; training: trained (underwent food safety training) or untrained (did not undergo any food safety training); area of training: good practices in food industry (GPFI) involving temperature monitoring, requirements for safe food, and so forth, work safety and hygiene (WSH) involving environmental hygiene, personal hygiene, requirements for protective wears, and so forth, or both; duration of training: short (<1 week), moderate (1-2 weeks), relatively long (3–6 weeks), and long (>6 weeks); and refresher training received within the last one year: yes (received) or none (none received).

Knowledge assessment section consisted of 10 questions while seven questions were constructed for the practice section. Some of the questions asked were in the aspects of food handling, hand washing during food preparation, working area, wearing of protective apron, and protecting open wounds, among others. Respondents were required to choose an option from a list of options for each question in the different sections. Each correct response was scored 1 and incorrect response 0. The marks were converted to 100% and classified as poor (marks below 50%), acceptable (marks between 50 and 74.99%), and excellent (marks ≥ 75%). This grading system has been found appropriate and useful for studies related to assessment “of food handlers” knowledge and practices [18].

Data were analyzed using Stata 12.0 (StataCorp LP, Texas, USA). Chi-square test was used to determine the relationship between food safety training, area of training, training duration, and refresher training and the knowledge and practice levels. In addition, variables on refresher training significant at the 10% significance level were included in the multivariate logistic regression model to determine the predictor variables for food handlers' knowledge and practice levels. The odds ratios were reported with their 95% confidence intervals (CI).

## 3. Results

### 3.1. Sociodemographic Characteristics

A total of 211 respondents participated in the study. Of these, 70.1% (148) were males and 10.4% (22) were within age group less than 20 years; 61.1% (129) were between ages 20 and 40 years while 28.4% (60) were above 40 years with the mean age being 35.5 years. Based on the level of education, 24.6% (52) had no formal education, 33.2% (70) had primary education, and 42.2% (89) had postprimary education. Almost 83% (175) had never received any training while only 17.1% (36) had training regarding food safety. Out of the trained food handlers, 25% trained in good practices in food industry; 41.7% trained in work safety and hygiene while 33.3% had their training in both areas. In addition, 44.4%, 16.7%, 8.3%, and 30.6%, respectively, had short, moderate, relatively long, and long training while 55.6% had refresher training. Almost 39% (14) and 61.1% (22) were from foodservice establishments with staff strength ranging from 5 to 10 to 20 and above, respectively.

### 3.2. Knowledge

As indicated in Table 1, there was a significant association between training and knowledge level (*χ* ^2^ = 26.38; *P* = 0.000) with higher proportions of trained foodservice workers demonstrating excellent (30.6%) and acceptable (38.9%) knowledge than those without training. The majority of the food handlers without food safety training had poor knowledge in most aspects of the questions asked. Most of them (95.4%) did not know what the term hazard analysis and critical control points (HACCP) meant and only 10.9% were aware that food handlers, in addition to raw food and insects, could also be a source of contamination to food. Moreover, 92.6% did not know that people with open skin injury, gastrointestinal disturbances, and eye/ear diseases should not be allowed to handle food to avoid contamination. Nevertheless, 62.3% knew that high temperature was the safe method to destroy bacteria (Table 2).

On the other hand, food handlers with food safety training showed good knowledge in most aspects except in their knowledge of the term HACCP, where only 30.6% gave the correct answers, and food storage temperature where 83.3% did not know that incorrect storing temperature of the refrigerator could increase the risk of food contamination (Table 2). The duration of training (*χ* ^2^ = 16.07; *P* = 0.01) was significantly associated with knowledge (Table 3). In addition, refresher training (*χ* ^2^ = 15.58; *P* = 0.000) was significantly associated with knowledge levels, being about 45 and 25 times more likely to, respectively, improve the knowledge level among food handlers with excellent (OR = 45; 95% CI: 3.47–584.34) and acceptable (OR = 45; 95% CI: 2.36–264.80) knowledge than those with poor knowledge (Table 4). However, area of training (*χ* ^2^ = 6.36; *P* = 0.17) was not significantly associated with the knowledge levels of food safety among the trained food handlers (Table 3).

### 3.3. Practice

There was a significant association observed between the foodservice workers' practice levels (*χ* ^2^ = 5.60; *P* = 0.05) and training, with significantly higher proportions of trained (30.6%) than the untrained (14.3%) foodservice workers demonstrating excellent practices (Table 1). Most of the food handlers with training had excellent and acceptable practices in the majority of food safety aspects such as cleaning the working area (61.1%), hand washing (58.3%), the use of potable water during food preparation (88.9%), and food storage at appropriate temperature (94.4%). However, only less than half (48%) would not handle food when they were ill particularly due to gastroenteritis, cough, or skin diseases (Table 5). On the other hand, the food handlers without food safety training though reportedly used potable water during food preparation (96.0%) and stored food at appropriate temperature (97.7%); they had lower score levels in major areas of food safety practices such as washing aprons (17.7%), cleaning work areas (16.0%), and hand washing (48.6%) (Table 5). Furthermore, the duration of training among trained foodservice workers was significantly associated with practice level (*χ* ^2^ = 22.33; *P* = 0.001) (Table 3). Refresher training (*χ* ^2^ = 11.50; *P* = 0.003) was also significantly associated with practice levels, being about 13.5 and 10.5 times more likely to, respectively, improve the practice level among food handlers with excellent (OR = 13.5; 95% CI: 2.01–90.69) and acceptable (OR = 45; 95% CI: 1.51–72.81) practice than those with poor practice (Table 4). Although food handlers who trained in both GPFI and WSH had higher practice score levels, the difference in areas of training (*χ* ^2^ = 8.49; *P* = 0.075) was not significantly associated with food safety practice levels (Table 3).

## 4. Discussion

The results of this study suggest that refresher and short duration training of not more than two weeks at a stretch are key features of an effective training programme for improved food safety practices. It was, however, observed that the area of training appeared not to have any significant impacts on the food safety knowledge and behaviour of the food handlers. Our findings suggest that refresher training and short duration training in addition to previously reported determinants [19–21] are essential to prevent food safety failures that often result from poor knowledge and practices of food safety among food handlers. Though earlier reports stated that increased knowledge from food safety training might not necessarily translate into improved attitudes and practices of food safety [10, 21, 22], our findings suggest that improved behaviour could be enhanced through the provision of regular refresher training to food handlers.

Our results further provide insights into several studies that reported inconsistent results regarding the effectiveness of food safety training on behaviour in foodservice establishments. Notably, these studies did not capture data on whether or not the respondents received refresher training. In addition, a large amount of time could have elapsed between the training received by some of the respondents and the questionnaire which might consequently hinder accurate responses from participants due to recall issues [23]. This might therefore account for the varying results obtained by the previous workers on the effectiveness of food safety training on knowledge and behaviour.

Findings throughout this study indicated a significant association between refresher training and the knowledge as well as practice levels of food handlers. Generally, regular refresher training provides more opportunities for food handlers to rehearse and update the skills earlier learnt. Our finding is further buttressed by the transfer of training theory which maintained that formal training period should be followed by additional learning opportunities [24, 25] which refresher training often provides. According to Worsfold et al. [26], the more opportunities the trainees have to use and rehearse these skills, the greater the probability that skills would be maintained, resulting in behaviour change and positive increments in job performance. As previously suggested, transfer of training into behaviour requires continual training, as the food handlers could have forgotten some of the essential skills learnt [20]. This assertion was substantiated by the knowledge survey conducted for the Food Standards Agency [27] in which only a minority of food handlers could remember all the important food hygiene practices in their workplace and the reasons for recommended storage, cooking, and cooling practices. Unfortunately, not many small food establishments have been providing refresher trainings [28], whereas management support that provides regular refresher training for food handlers would help reinforce the adoption of safe food handling behaviours [20].

Furthermore, there was a significant decline in the knowledge and practice levels of the trained food handlers with increasing duration of training received. This observation suggests that the prolonged training session might not necessarily guarantee increased knowledge and improved food safety behaviour. Prolonged training despite training contents and other related factors could result in lower returns given the possibility of redundancy and boring repetitiveness. From his findings, Yang [29] reported dissatisfaction of food hygiene trainees who underwent prolonged training and recommended that short duration training would be better. This worker further indicated that the trainees maintained that the contents of the training were repetitive. Besides, the ability of food handlers in general to cope with prolonged training differs, resulting in different levels of assimilation and consequent behaviour. This assertion is supported by the transfer of training theory which indicated that cognitive ability is crucial for the transfer of training with those with higher cognitive ability being able to successfully acquire, comprehend, and utilize the training competencies [11]. Although this ability is not a characteristic that foodservice industry can necessarily control, considering this will help in determining which foodservice employees should attend which training with reference to its required duration. Generally, training duration should be relatively short, not more than two weeks at a stretch, in order to enhance optimal food safety knowledge and behaviour among food handlers. Therefore, training duration should be considered when examining the association between training and food handlers' knowledge and behaviour.

The results of this study revealed that area of training did not have any significant association with the knowledge and practice levels of the food handlers. As observed, the food handlers who trained in good practices in food industry as well as in work safety and hygiene did not have significantly higher score levels than those who trained in either of the two areas. This therefore suggests that food safety training courses should focus specifically on the area of work or need of each food handler rather than subjecting every food handler to various training areas that might not be relevant to their ultimate improved performance. More so, the needs of individual food handlers are likely to vary considerably in what they need to be trained on. This finding is consistent with previous observations [22, 26, 30] which indicated that many people perceived accredited basic or foundation level food hygiene training as not being relevant to the whole food industry. In addition, focusing food safety training on the specific need or work area of food handlers would save time, money, and resources.

### 4.1. Limitations

This work had some limitations. The number of trained food handlers was relatively small when compared to the proportion of those who had not received any training. Higher number of trained food handlers could have possibly provided better insights into the impacts of refresher training, training duration, and area of training on the knowledge and practice levels of the food handlers. Again, the number of food handlers interviewed in all was relatively small. The authors feel confident, however, that information gained from this population could generalize to other food handlers in the study area.

## 5. Conclusions

This study is considered an important step in increasing our understanding of the role of different food safety training components in determining the effectiveness of training on food handlers' knowledge and behavior. Refresher and relatively short duration training remain essential for improved food safety behaviour among foodservice workers. The findings indicate that prolonged training duration does not necessarily connote increased knowledge gain or improved behaviour. Rather, improved performance could be achieved when training duration is relatively short. Furthermore, training in all areas of food safety does not imply improved performance in food safety issues; training contents should be directed toward the specific need of the individual food handlers and their area of work in the food industry. Overall, researchers should always consider varying training components before making assertions regarding effectiveness of training on foodservice workers' behaviour.

## Figures and Tables

**Table 1:** Food safety knowledge and practice scores among foodservice workers with and without training (*N* = 211).

Variables^*^	Total			Trained (*N* = 36)			Untrained (*N* = 175)			*χ* ^2^; *P* value
	Excellent	Acceptable	Poor	Excellent	Acceptable	Poor	Excellent	Acceptable	Poor	
	*n* (%)	*n* (%)	*n* (%)	*n* (%)	*n* (%)	*n* (%)	*n* (%)	*n* (%)	*n* (%)	
Knowledge	19 (9.0)	59 (28.0)	133 (63.0)	11 (30.6)	14 (38.9)	11 (30.6)	8 (4.6)	45 (25.7)	122 (69.7)	26.38; 0.000
Practices	36 (17.1)	76 (36.0)	99 (46.9)	11 (30.6)	9 (25.0)	16 (44.4)	25 (14.3)	67 (38.3)	83 (47.4)	5.60; 0.05

^*^Poor (marks below 50%), acceptable (marks between 50 and 74.99%), and excellent (marks ≥75%).

**Table 2:** Food safety knowledge levels among foodservice workers with and without training.

Statements	Trained (*N* = 36)	Untrained (*N* = 175)
	Yes (%)	Yes (%)
Heard of the term HACCP	30.6	4.6
Food handlers, raw food, and insects can be a source of contamination to food	69.4	10.9
People with open skin injury, gastrointestinal disturbances, and eye/ear diseases should not be allowed to handle food to avoid contamination	55.6	7.4
Wound protection during food handling would prevent food contamination	75.0	45.7
An incorrect application of sanitizers can increase the risk of food-borne illness to consumers	61.1	14.3
Washing and cleaning of working surfaces can reduce contamination of food	66.7	49.7
Washing hands before and in-between food handling reduces contamination	69.4	37.7
High temperature is the safe method to destroy bacteria	61.1	62.3
The correct temperature of water in sterilizers for knives is 82°C	55.6	7.4
Incorrect storage temperature of the refrigerator can increase the risk of food contamination	16.7	4.0

**Table 3:** The relationship of food safety training variables and foodservice workers' knowledge and practice levels.

Variables	Knowledge^**^				Practices^**^				Total (%)
	Excellent	Acceptable	Poor	*χ* ^2^; *P* value	Excellent	Acceptable	Poor	*χ* ^2^; *P* value	
	*n* (%)	*n* (%)	*n* (%)		*n* (%)	*n* (%)	*n* (%)		
Area of training									
GPFI^*^	5 (55.6)	2 (22.2)	2 (22.2)	6.36; 0.174	0 (0.0)	3 (33.3)	6 (66.7)	8.49; 0.075	9 (25.0)
WSH^†^	3 (20.0)	5 (33.3)	7 (46.7)		4 (26.7)	4 (26.7)	7 (46.6)		15 (41.7)
Both	3 (25.0)	7 (58.3)	2 (16.7)		7 (58.3)	2 (16.7)	3 (25.0)		12 (33.3)
Duration of training (in weeks)									
<1	6 (37.5)	9 (56.3)	1 (6.3)	16.07; 0.012	8 (50.0)	5 (31.2)	3 (18.8)	22.33; 0.001	16 (44.4)
1-2	3 (50.0)	2 (33.3)	1 (16.7)		0 (0.0)	4 (66.7)	2 (33.3)		6 (16.7)
3–6	1 (33.3)	0 (0.0)	2 (66.7)		1 (33.33)	0 (0.0)	2 (66.7)		3 (8.3)
>6	1 (9.1)	3 (27.3)	7 (63.7)		2 (18.2)	0 (0.0)	9 (81.8)		11 (30.6)
Refresher training									
Yes	9 (45.0)	10 (50.0)	1 (5.0)	15.58; 0.000	9 (45.0)	7 (35.0)	4 (20.0)	11.50; 0.003	20 (55.6)
None	2 (12.5)	4 (25.0)	10 (62.5)		2 (12.5)	2 (12.5)	12 (75.0)		16 (44.4)

^*^GPFI: good practice in food industry.

^†^WSH: work safety and hygiene.

^**^Poor (marks below 50%), acceptable (marks between 50 and 74.99%), and excellent (marks ≥75%).

**Table 4:** Logistic regression predicting food handlers' refresher training with knowledge and practice levels as independent variables.

Variable^*^	Refresher training		*P* value
	OR	CI	
Knowledge			
Poor	1.0 (reference)		
Acceptable	25	2.36–264.80	0.008
Excellent	45	3.47–584.34	0.004
Practice			
Poor			
Acceptable	10.5	1.51–72.81	0.017
Excellent	13.5	2.01–90.69	0.007

^*^Poor (marks below 50%), acceptable (marks between 50 and 74.99%), and excellent (marks ≥75%).

**Table 5:** Food safety practice level among foodservice workers with and without training.

Statements	Trained (*N* = 36)	Untrained (*N* = 175)
	Yes (%)	Yes (%)
I wash my aprons after each day's work.	55.6	17.7
I always clean the work area before and after work.	61.1	16.0
I wash my hands before I start work.	58.3	48.6
I use potable water during food preparation.	88.9	96.0
I do not handle food with unprotected wound.	52.8	63.4
I do not handle food when I am ill especially due to gastroenteritis, cough, or skin diseases.	48.0	54.9
I always store food at appropriate temperature.	94.4	97.7
